# A city-wide examination of fine-grained human emotions through social media analysis

**DOI:** 10.1371/journal.pone.0279749

**Published:** 2023-02-01

**Authors:** Panote Siriaraya, Yihong Zhang, Yukiko Kawai, Peter Jeszenszky, Adam Jatowt

**Affiliations:** 1 Faculty of Information and Human Science, Kyoto Institute of Technology, Kyoto, Japan; 2 Multimedia Data Engineering Lab, Osaka University, Osaka, Japan; 3 Faculty of Computer Science and Engineering, Kyoto Sangyo University, Kyoto, Japan; 4 Osaka University, Osaka, Japan; 5 University of Bern, Bern, Switzerland; 6 Digital Science Center and Department of Computer Science, University of Innsbruck, Innsbruck, Austria; Beihang University, CHINA

## Abstract

The proliferation of Social Media and Open Web data has provided researchers with a unique opportunity to better understand human behavior at different levels. In this paper, we show how data from Open Street Map and Twitter could be analyzed and used to portray detailed Human Emotions at a city wide level in two cities, San Francisco and London. Neural Network classifiers for fine-grained emotions were developed, tested and used to detect emotions from tweets in the two cites. The detected emotions were then matched to key locations extracted from Open Street Map. Through an analysis of the resulting data set, we highlight the effect different days, locations and POI neighborhoods have on the expression of human emotions in the cities.

## Introduction

The pervasive growth of social media has resulted in a large amount of digital information becoming available in the past decade. As a result, it has become possible to obtain near real-time information about the opinions, activities and behaviors of various population groups by automatically analyzing data from public social network services such as Twitter and Flickr [1]. This trend has allowed researchers to better understand patterns of human behavior at an individualized level, but yet in a macroscopic scale. Notable examples include prior studies which have used social media data to highlight emerging topics and trends [2] or to provide an overview of the mobility patterns for an entire population [3, 4].

In recent studies, researchers have begun to investigate whether data from social media sources could also be useful in portraying the implicit aspects and characteristics of users (such as their emotions) in addition to their explicit behaviours (such as their activities and movement patterns). Notable examples include the “pulse of the nation” project, which provides a broad visualization of happiness across the USA [5]. In most of these studies however, emotions were often matched to a country, state or city level spatial resolution, thus limiting the scope of possible research questions which could be addressed through the data set [6, 7]. For example, while it is possible to carry out large scale analysis to correlate the overall emotional status of the community population with measures related to well-being (such as in [8]), it is difficult to investigate relations at a more detailed spatial level, such as the role different recreational facilities (bars, sport venues, temples etc.) might have on mood and emotion in a specific city. In addition, earlier works also tended to focus primarily on positive or negative sentiment measures [9] and not on specific emotions.

In this study, we expand upon the previous research domain and utilize data from the social media platform Twitter to analyze fine-grained human emotions at a city wide level. Different approaches for automatically detecting fine-grained human emotions from the social media platform Twitter were examined and evaluated. Afterwards, Neural Network classifiers were used to identify emotions from geotagged tweets in two cities, San Francisco and London and data from Open Street Map data was used to spatially contextualize the social media information. Finally, we use the resulting data set to (1) *Visualize the temporal characteristics of emotions within the two cities* and (2) *Analyze the influence different place types have on the expression of emotions*. Overall, the aim of our paper is to show how it is possible to study the characteristics of fine-grained emotions at both a spatial and temporal level throughout the whole city, using current technology and publicly available data sets. Our research is unique in that whereas prior studies tend to focus on analyzing sentiment polarity or a single emotion such as happiness, we examined seven different fine-grained emotions (Anger, Anticipation, Disgust, Fear, Trust, Joy and Sadness) at a city-wide level based on large-scale data analysis from social media. In addition, we also examine how these emotions are influenced at a more detailed spatial level (i.e. at a Point of interest level), as opposed to previous studies which tend to analyze data at coarse geographic levels (at the state, city or country level etc.). The results from this study would serve as a preliminary basis for future research looking to investigate the occurrence of such emotions through large scale social media analysis.

This paper is structured as follows. First, we describe how spatial and social media data from Open Street Map and Twitter was collected and processed for San Francisco and London. Next, we highlight the results of an experiment study carried out to evaluate different classification algorithms which could be used to detect user emotions from geotagged tweets, the best performing ones were then used to analyze the tweets collected in this study. Afterwards, we analyzed the characteristics of the daily level of emotions exhibited by the population in the two cities. Finally, we investigate the potential effect different place types have on each of the seven user emotions, first by examining the degree of emotions exhibited by users at different locations and then examining how the display of emotions is influenced by the type of locations at different vicinity levels.

## Related research

The use of social media data to analyze and provide a macro scale display of human emotions has been a popular topic of research in past studies. Earlier works tended to focus on broad sentiment measures such as positive and negative emotions. In such studies, sentiment analysis is generally carried out on geotagged tweets to provide an overview about the overall mood of the population at different regions or cities [10]. Other studies focused on identifying user sentiment during or after a particular event (such as a pandemic [11, 12] or terrorism event [13]) or analyzed user sentiment at different geographical areas with the aim of investigating the mobility patterns of people within a city [14]. In a number of these studies, data about the sentiment of the tweets themselves were correlated with various metrics affecting urban living (such as job opportunities and access to public transportation) to provide insight into the effect these measures have on the happiness of the population [15]. In a more applied setting, researchers have also incorporated sentiment analysis data from geotagged social media as part of a larger system or predictive model to recommend safe walking routes [16] or improve the accuracy of crime prediction [17].

More recent studies have begun to explore the expression of detailed emotions (such as fear and anger) through geotagged tweets. For instance, in the “We feel project”, researchers highlighted how people in different regions and countries exhibited the five primary emotions using geotagged tweets [18]. In another example, geotagged tweets were classified based on emotional dimensions such as pleasantness and dominance, the results of which were visualized over the world map to show emotional trends across different countries [19]. Other studies used geotagged tweets to analyze the emotions of people (fear, sadness, sympathy) in response to particular events such as the London Westminster and London Bridge attacks [13] and the Paris attacks (anger and sadness) [20].

These earlier studies which were discussed had several limitations however. First, sentiment mapping is generally carried out at a country or state wide level and tends to focus on polarized sentiment measures (varying degrees of positive and negative emotions) and not on specific emotions (such as Anger, Joy and Disgust). While such studies are particularly useful for visualization purposes and to help researchers understand general trends regarding the mood of entire population groups, a higher resolution analysis into user emotions at a street or POI level would allow researchers to address more detailed questions about the city, such as the effect specific locations or places might have on the behaviour and emotions of people. A number of studies have suggested how this could be the case. For example, it is well known that green spaces within a city could contribute to an increase in positive mood and emotions [21]. In addition, the various locations within a city could elicit different emotional responses (arousal, tension etc.) for people walking along the street [22]. Studies have also shown that people near locations such as transportation hubs and sewage facilities tend to report more negative emotions and those near landmark buildings and tourist locations tend to report more positive ones [10]. Overall, this type of analysis could be extremely useful in fields such as urban planning and tourism and there have been more calls for further research to be carried out in this area of urban emotions [23]. We contribute to this direction of research in this paper, by showing how seven different fine-grained emotion types could be analyzed at a city-wide level. In addition, we provide an analysis into the effect of different place types have on human emotions at a POI level.

## Data collection

In this study, data collection was focused on two cities, San Francisco in the USA and London in the UK. The main reasons why these two cities were selected were: 1) The dense open data footprint (social media and open data) which was available (i.e. there were sufficient numbers of geotagged tweets, and Open Street Map was generally found to be more accurate in such densely populated urban areas [24]) and 2) The majority of the data was available in English, which was necessary as we were only able to assemble a sufficient number of training samples in that language when developing the automated models used to classify the different emotions. Data from Open Street Map was collected and used to represent the spatial characteristics of the city, while data from geotagged tweets was analyzed and used to represent the emotions of people within the city. When collecting data for this study, we complied fully with the terms and conditions for the data sources.

### Collecting spatial information

One notable source of information about the characteristics of the various locations and places in the physical world is Open Street Map (OSM) [25] This platform was created to provide a free open and editable map of the world, where information is provided by volunteers who collaborate with each other to generate knowledge about the streets, buildings and locations which exist in various cities. Despite OSM data being generated primarily by volunteers, studies show that the quality of the data available from this platform compares quite favorably with traditional spatial data sources, particularly in dense urban areas [24] (however, in underpopulated or rural areas, data could be less accurate [26, 27]).

In this study, OSM tags from the nodes, ways and relations were used to represent the characteristics of different locations in the city. For example, a pet shop is generally tagged using a “key”:“value” format of “shop”: “pet”. The OSM elements which contain appropriate tags (such as leisure, shop or amenity tags) were considered as Points of Interests (POI). Table 1 shows the number of OSM elements and Points of Interests extracted for San Fransisco and London. As users could freely edit the tags in the OSM data, the dataset resulted in a large number of redundant tags which represented similar objects (such as dry_cleaners and laundry etc.). This made it difficult to differentiate between meaningful categories. In addition, if these tag value were used as categories for the POIs, they would be too detailed for conducting analysis (e.g. for the tag values identified in the cities of San Francisco and Greater London, less than 47% contained more than 3 locations in each city and thus when matching tweets to the POIs based on tag values, a large proportion of POIs would have few or no tweets in the vicinity). Therefore, the tags in the dataset were further classified into categories proposed by the Ordnance Survey Classification scheme in the UK [28] This classification scheme contains nine categories (Hotel and Restaurants, Commercial Services, Attractions, Sports and Entertainment, Education and Health, Public Infrastructure, Manufacturing and Production, Retail, Transportation). In addition, we also added two other groups not included in the classification scheme, Residential to denote locations which are marked as places of residence and Office to denote workplaces. Residential locations are often tagged in OSM with values such as “flat”, “house” or “apartment”, while office buildings generally contain tags such as “office building”. When pre-processing the tags, tags which were of the same category but were written differently were also merged (such as those which were different due to singular and plural notations) or were different due to being written in American or British English (such as flat/apartment).

**Table 1:** The OSM data in the areas used in our study.

Area	OSM-elements	POI	Unique POI Types
Greater London	4,823,654	236,482	1,425
San Fransisco	3,211,795	21,816	527

Overall, there were 1,425 Unique POI types identified from the POI locations in Greater London and 527 Unique POI types identified from the POI locations in the city of San Francisco. To classify these POI types into the categorizes proposed by the Ordinance Survey classification scheme, one researcher familiar with the scheme read through each of the Unique POI types and classified them into one of the 11 categories by hand. For example, the POI type “fast_food” was classified as part of the “Hotel and Restaurants” category and “clothes” was classified as part of the “Retail” category. When the POI type was ambiguous, the researcher referred back to several of the raw locations that used the POI type and examined the names and meta data of those locations (which could contain information such as the opening time, website url etc.) and referred to the data on OpenStreetMap’s wiki page [29] to make a decision. Furthermore, another researcher sampled the classified categories (especially those which were ambiguous) and any differences were verbally discussed and agreed upon.

Overall, there was a considerable difference between some of the POI types which exist within the two cities. For example, a larger proportion of the POIs (57.52%) in the London dataset was tagged as a Residential while only (24.52%) of the elements in San Fransisco were tagged as such. Further examination of the Non-Residential and Non-Workplace tags also showed that while San Francisco contained more venues which serve food for customers (such as Restaurant & Cafes) (26.70%) compared to London (18.75%), the proportion of Retail Stores which sell food items was more than double compared to London (12.37%) than in San Francisco (5.34%). Other notable differences were in the proportion of Attractions (tourism spots, recreational facilities) in San Francisco (23.48%) when compared to Greater London (16.59%).

### Collecting geotagged tweets

Prior studies have shown how data from Twitter could be particularly useful in helping researchers understand human behavior and emotions. The public availability of information from this platform combined with the high frequency in which users provide information about the world around them has allowed Twitter to become a valuable data source in a number of data analysis studies [1, 18].

In this study, geotagged tweets were used in a similar manner as previous studies: as a proxy to explore human emotions at specific locations. Geotagged tweets were collected from the cities of London and San Francisco for a one year period, from 1/9/2016 to 28/8/2017 (which coincided with the year of the tweets used to train our models to detect emotions). We chose to use geo-tagged tweets in the earlier years in our analysis due to the more accurate location data [30]. Similar to other studies of this nature [31–33], the geotagged tweets were collected using the Twitter Streaming API. A location query was used, with the bounding box approach to gather geotagged tweets within the areas of San Francisco and Greater London (e.g. we defined the latitudes and longitudes of the Northwest and Southeast Corners of a rectangular region and all tweets that are within that region would be collected. For San Francisco, this region was set to Southeast Corner: (37.7020,-122.3362) Northwest Corner: (37.8355,-122.5422) and for London, this region was set to Southeast Corner: (51.2765, 0.3571) Northwest Corner: (51.686,-0.5713)). In addition to the tweet text, we also collected meta data such as the user id, latitude, longitude, date and time of the tweet and language of the tweet. Overall, we collected approximately 0.96 million tweets in San Francisco and 2.18 million tweets in Greater London. In the pre-processing stage, the content of the tweets were examined and tweets which were automatically generated using linked applications (such as Foursquare) were identified using regular expression matching and were removed. These tweets generally do not contain content posted by actual users, mostly showing messages such as “I’m at X Location” tweets, “Just posted a photo/video @X” tweets, “Tmp 20C Wind 0mph Press 1010.0mb Cloud 1538 ft Rain 6.7mm Humidity 79” (which are automatically generated by weather stations). After pre-processing, there were 1.57 million tweets remaining for London (180,000 unique users) and 0.39 million tweets for San Francisco (65,000 unique users).

## Detecting basic emotions

Although the task of analyzing sentiment polarity from tweets (positive and negative etc.) has been well explored in prior studies ([9, 34] etc.), research into systems which allow us to detect the presence of more specific emotions in text based contexts have only emerged in recent years [35]. In this study, we focus on identifying the presence of the eight emotion types proposed by Plutchik’s multi-dimensional wheel of emotions (Anger, Anticipation, Disgust, Fear, Joy, Sadness, Surprise, Trust) in the geotagged tweets [36]. A binary classifier was constructed for each of the eight different emotions which would determine whether or not each tweet displays that particular emotion. We then examined the performance of six different classification approaches for detecting emotions from the tweets.

1. **SVM+Glove**: The pre-trained GloVe model (the 200 dimension Global Vectors for Word Representation trained with 22 billion tweets [37] was used to vectorize the tweets and the classification was performed using the SVM (Support Vector Machine) model with the mean value of the vectorized tweets as features. For SVM models, we experimented with different parameters and kernels and decided to use a linear kernal SVM with a C value of 1 and hinge loss function.
2. **SVM+Lexicon**: A lexicon based approach was used to train the model (see [35, 38, 39]), in which several word lexicons were used to extract the emotional and sentiment features from each tweet. Overall, 25 different lexicons were used as features to train the SVM model. We experimented with different combinations of lexicons (from a total of 35 different lexicons) and the 25 lexicons combination used in this study resulted in the best performance. The lexicons which were used include the NRC-10 word-emotion association expanded lexicons and the NRC-10 Hash-emotion association lexicons (emotion lexicons for hash tag data) for the eight basic emotions as well as the positive and negative SentiWord and emoticons lexicons.
3. **SVM+Lexicon+Glove**: The vectorized tweets (Glove) and the extracted lexicon based emotions (similar to the SVM+ Lexicon model) were both used as input data.
4. **NN+Glove**: The vectorized tweets were classified using a Neural Network (2 layer dense Neural Network with 200 nodes sequentially connected. The last layer utilized a sigmoid activation function).
5. **LSTM+Glove**: The vectorized tweets (Glove) were classified using an LSTM (Long short-term memory) neural network model (2 layer LSTM with 200 nodes and a drop-out layer of 0.4 sequentially connected to each other, the last layer utilized a sigmoid activation function). For the LSTM models, an RMSprop opitmizer was used with the binary cross-entropy loss function.
6. **Hybrid+Lexicon+Glove**: The vectorized tweets (Glove) and the extracted lexicon based emotions (similar to the SVM+ Lexicon model) were used as input in a hybrid Neural Network model. The Neural Network structure which had been used previously in [40] for bot detection was adapted and applied instead to detect emotions. S1 Fig shows the architecture of this model.

The data used to train our models were obtained from the SemEval2017 and 2018 dataset. Overall, this dataset contained approximately 11,700 labeled tweets from 2016 to 2017. The SemEval dataset was used to evaluate and train our models as it contained a sufficiently large number of human-labeled geotagged tweets which originated from the same time period as the tweets collected and used in our study. Evaluation was carried out using 10-fold-cross validation. The dataset was shuffled randomly and split into 10 groups with each unique group being used as the testing data and the remaining nine groups being used as the training data. The F-score was used as the evaluation score in the 10-fold-cross-validation. This score takes into account both Precision (the items correctly classified in each category) and Recall (the percentage of the category that was successfully classified) and is usually considered as more suitable for evaluating classifier systems than just a measure of overall accuracy.

**Fig 1:**
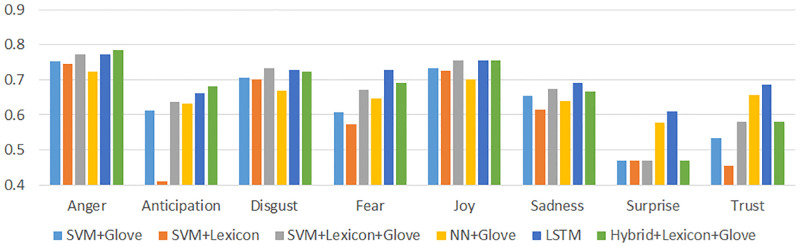
10-fold cross validation F-score of the six different classifiers for the eight basic emotion types.

**Fig 2:**
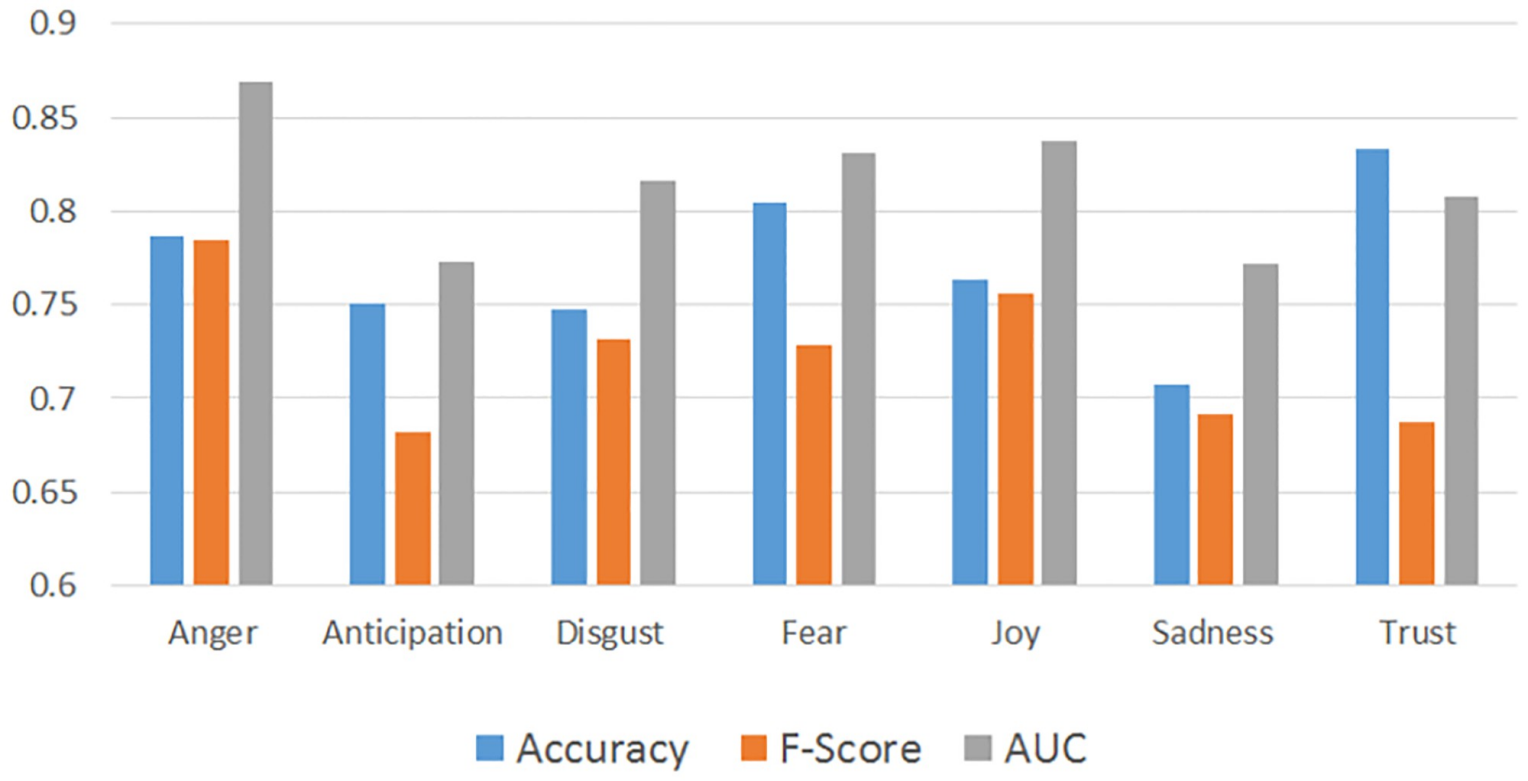
Performance metrics for the models used in the study (10-fold cross validation).

Fig 1 shows the results of this evaluation. Overall, the **Hybrid+Lexicon+Glove** performed best in classifying emotions of Anger and Anticipation and the **LSTM+Glove** model performed best in classifying Fear, Joy, Sadness, Surprise and Trust. Finally, the **SVM+Lexicon+Glove** performed best in classifying the emotion of Disgust. In the proceeding analysis, the models which showed the best results were used to classify their respective emotions for this study. However, as Surprise performed quite poorly (obtaining an F-score of only 0.61 in the best case model), this emotion was excluded from the subsequent analysis. For comparison purposes, the performance of the models used in this study are summarized in Fig 2 based on Accuracy, F-Score and AUC.

Using the previously mentioned classifiers, we analyzed the fine-grained emotions of the tweets collected in the study. 3.1% of the tweets had been classified with the emotion Anger, 5.5% Disgust, 5.8% Anticipation, 49.2% Joy, 4.1% Sadness, 3.7% Fear and 6.1% Trust for the city of San Francisco. In London, 3.8% of the tweets had been classified with the emotion Anger, 5.9% Disgust, 4.8% Anticipation, 49.5% Joy, 4.6% Sadness, 2.8% Fear and 4.0% Trust. Table 2 (at the end of the manuscript) shows examples of the tweets which were classified for each emotion that were used in the study.

**Table 2:** Examples of tweets classified for each emotion in London and San Francisco.

Emotion	Tweet Content
Joy	Had a nice evening at the <Place Name>
	On beautiful days like today, if you can’t adjust your walking times for the cooler hours
	Amazing evening to celebrate <Person> birthday.
	Fashion show 2017 … great time
	That’s a wrap! Great day with great people.
Anger	How rude!
	40 minutes on hold to pay a £100 council tax bill no they can fuck off I’ve got to go back to work they can wait now!!Piss takers
	When I had a job that asked me to do some bullshit I quit. Personal integrity. All those drivers should quit
	Asshole on the bus is jovially yelling at his friend on the phone and spitting out the window. Stay classy San Francisco
	45 minutes stuck between stations on <Station name> this morning. Fuck you very much
Digust	Yucky stench of weed outside London <Station name>!
	This place smells like piss and beer can you get me out?
	URGH gross @<University>
	eew smells like a sewer this morning…
	Are weed products getting way too ridiculous or did I just get paid for a show in heroine?
Sadness	Sad to wave goodbye to <Place Name> It has been an amazing experience working there
	I’m sick with a cold. Headache, body aches, coughing, sore throat
	Just had to try it and instantly regretted that decision. Threw it away after a couple of sips.
	Flowers laid for those injuired and killed in Sunday’s attack.
Anticipation	I really wanna try fried chicken and waffles…
	Happy New Year 2017. Im sure its going to be awesome!
	Cant wait to be spending Fridays up on the rooftop. Definitely going to be a firm favourite.
	@<Person> we are coming to see <Music event> at the end of month. Its my fave musical ever so excited
	Here comes the brand new flava in year!
Trust	<Name> gives my lil’ Bug the seal of approval!
	Thank you @<Name> for providing one of the many high quality, premium baby products
	One of the most epic meals of all time!
	I appreciate quality and our team made some magic this weekend, so many great memories with <URL link to place website>

## Temporal examination of emotions

To examine the temporal characteristics of emotion, we first calculated the level of emotions exhibited by the population on each date. This was done based on the ratio of the number of tweets which the user had sent during that day that had been positively classified as exhibiting an emotion by our models. For example, if a user sent one angry tweet and 9 other non-angry tweets on a specific day, the angry emotion ratio for that user would be 0.1. Calculating the emotions for each user individually was necessary to prevent bias from individuals who tend to send a large number of tweets (the number of tweets sent by each user tended to be unevenly distributed to a considerable degree). Tukey’s fences were also used to filter out low range outlier days based on the number of tweets sent on that day (using K = 1.5) [41]. This helped filter out days where there were only few tweets, as days which had only a few users tweeting biased the average results (such days tended to only have a few users who tweeted one tweet that turned out to be positively classified; this would result in an abnormally large average). Afterwards, the emotion value for all users on that day were averaged resulting in the daily emotion value. A time series of the emotions displayed on each day is shown in S2 Fig for San Francisco and S3 Fig for London.

### Visualizing daily emotions throughout the year

**Fig 3:**
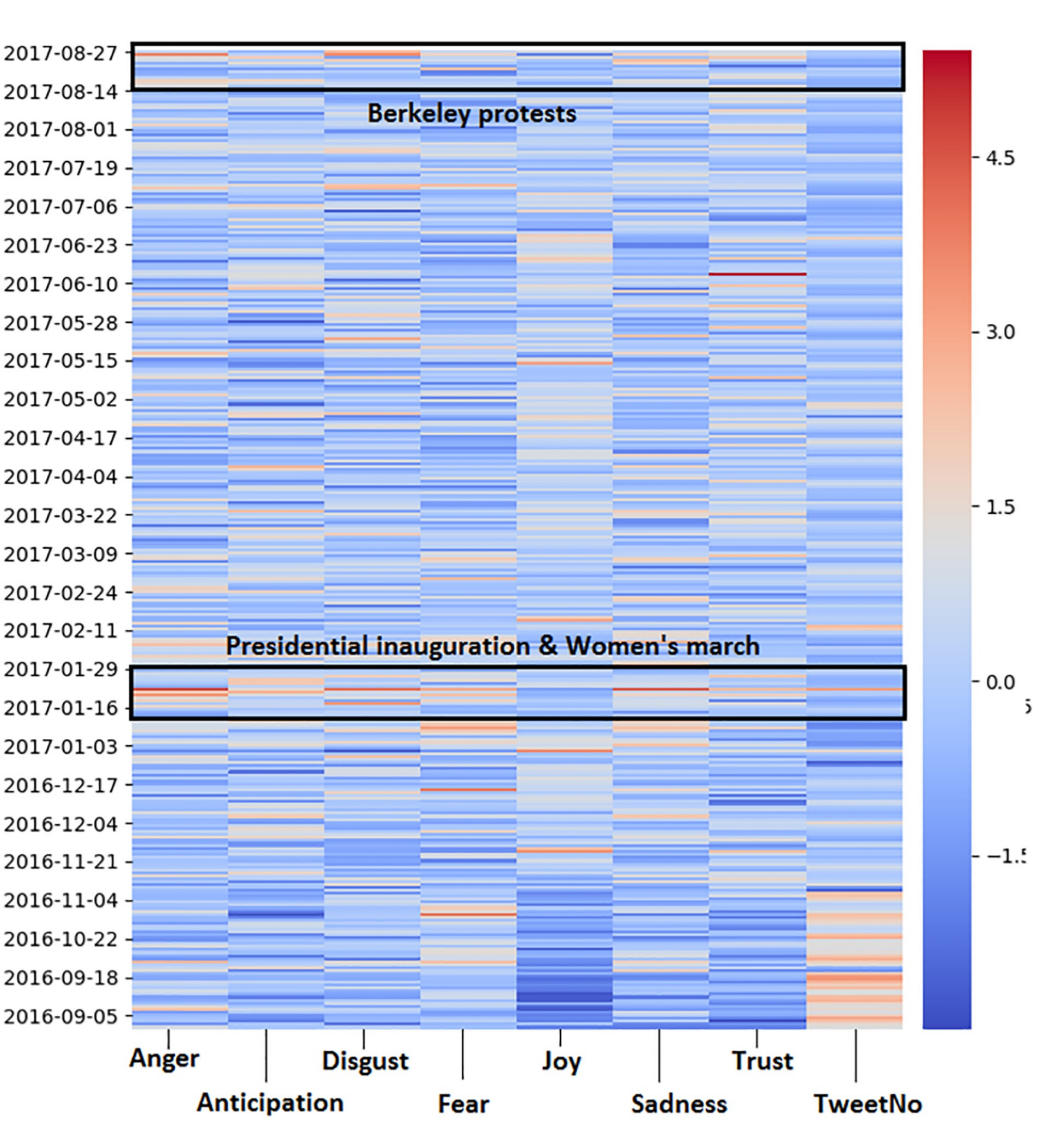
A time based heatmap of San Francisco (red = high levels of emotion, blue = low levels of emotion).

Temporal heat maps were created to visualize the average emotions on different days in San Francisco and London. The heatmap for the city of San Francisco is shown in Fig 3. It should be noted that the heatmap values were presented using the z-score, allowing for an easier comparison between the different emotions. A visual inspection of the values showed spikes in emotion levels during the period of 20 Jan 2017 to 22 Jan 2017. This coincided with two events in the U.S., the Presidential inauguration and the 2017 Women’s March. During the Women’s March in particular, users showed the highest level of Anticipation one day prior to the event (21 Jan 2017) (0.106 compared to a daily average of 0.07) and the highest level of Anger (0.069 compared to a daily average of 0.03), Disgust (0.097 compared to a daily average 0.058) and Sadness (0.081 compared to a daily average of 0.044) on the day of the event (22 Jan 2017). Another prominent event was the 2017 Berkeley protests that occurred during 26 Aug 2017, which resulted in the second highest level of Anger (0.615) and Disgust (0.092). On seasonal events such as New Years Eve, users showed the highest level of Joy (0.612 compared to a daily average of 0.502).

**Fig 4:**
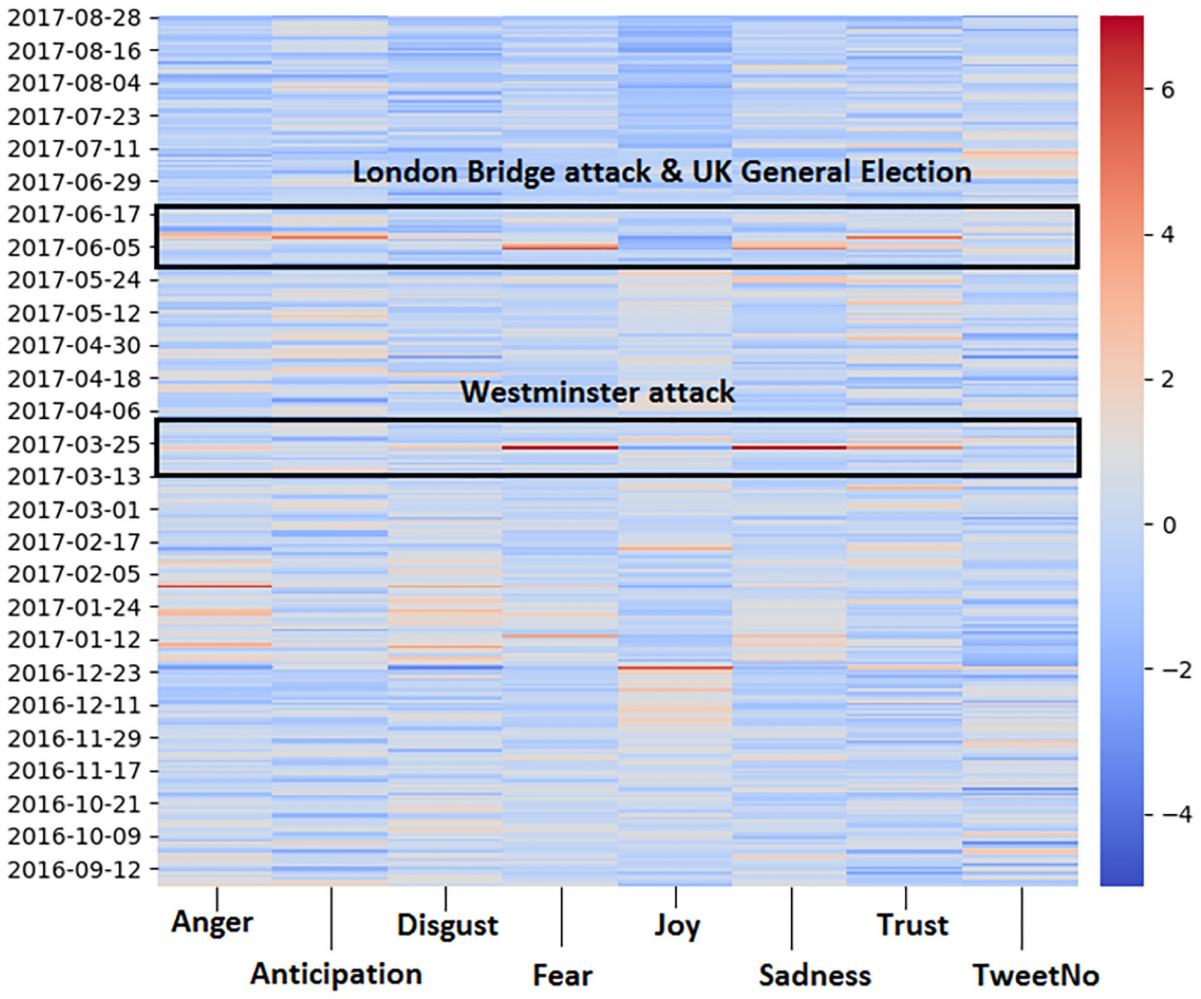
A time based heatmap of London (red = high levels of emotion, blue = low levels of emotion).

The heatmap for the city of London is shown in Fig 4. Overall, there were two events which have a high spike in emotion levels in London. These were the Westminster attacks which occurred around 23 March 2017 and the London Bridge attack which occurred during 3 June 2017. Twitter users in London showed the highest level of Fear during the Westminster attack (0.097 compared to a daily average of 0.0275) and the second highest level at the days following the London Bridge attack (0.067 at 4 June 2017)(0.053 at 5 June 2017). The highest level of Sadness also occurred during the Westminster attack (0.122 compared to a daily average of 0.04) and the second highest during the days directly after the London Bridge attacks (0.084 at 4 June 2017). Seasonal events also played a prominent role, with New Years showing high levels of Joy (0.636 compared to a daily average of 0.509) as well as Valentine’s Day showing the second highest level of Joy (0.581) and the Christmas week (around 0.55 on 23 December for example). The highest level of Trust (0.0635 compared to a daily average of 0.041) and Anticipation (0.092 compared to a daily average of 0.056) was shown during UK General election (8 June 2017).

Overall, these results were mostly consistent with those reported in previous studies. For instance, prior studies found that during events of terrorism, there tends to be a spike in the display of emotions such as fear, sympathy and sadness on social media such as twitter based on the geographic proximity of the user to that event [42] or in space-time clusters [13]. A similar result was also discovered when analyzing tweets during the Paris and Brussels attacks, where emotions such as Anger, Sadness and Anxiety tending to spike on social media a couple of days after the event [20]. In terms of positive emotion, prior studies which analyzed geotagged tweets also showed that users tended to display more Joy and Happiness on holiday events such as Christmas and Valentine’s day [43, 44]. However, the results from this study also adds to those from the earlier studies, showing how terrorism events could elicit other negative emotions such as Disgust and that high levels of emotions such as Anticipation, Anger and Disgust could also be found during events such as protests.

### Emotions on different days of the week

**Fig 5:**
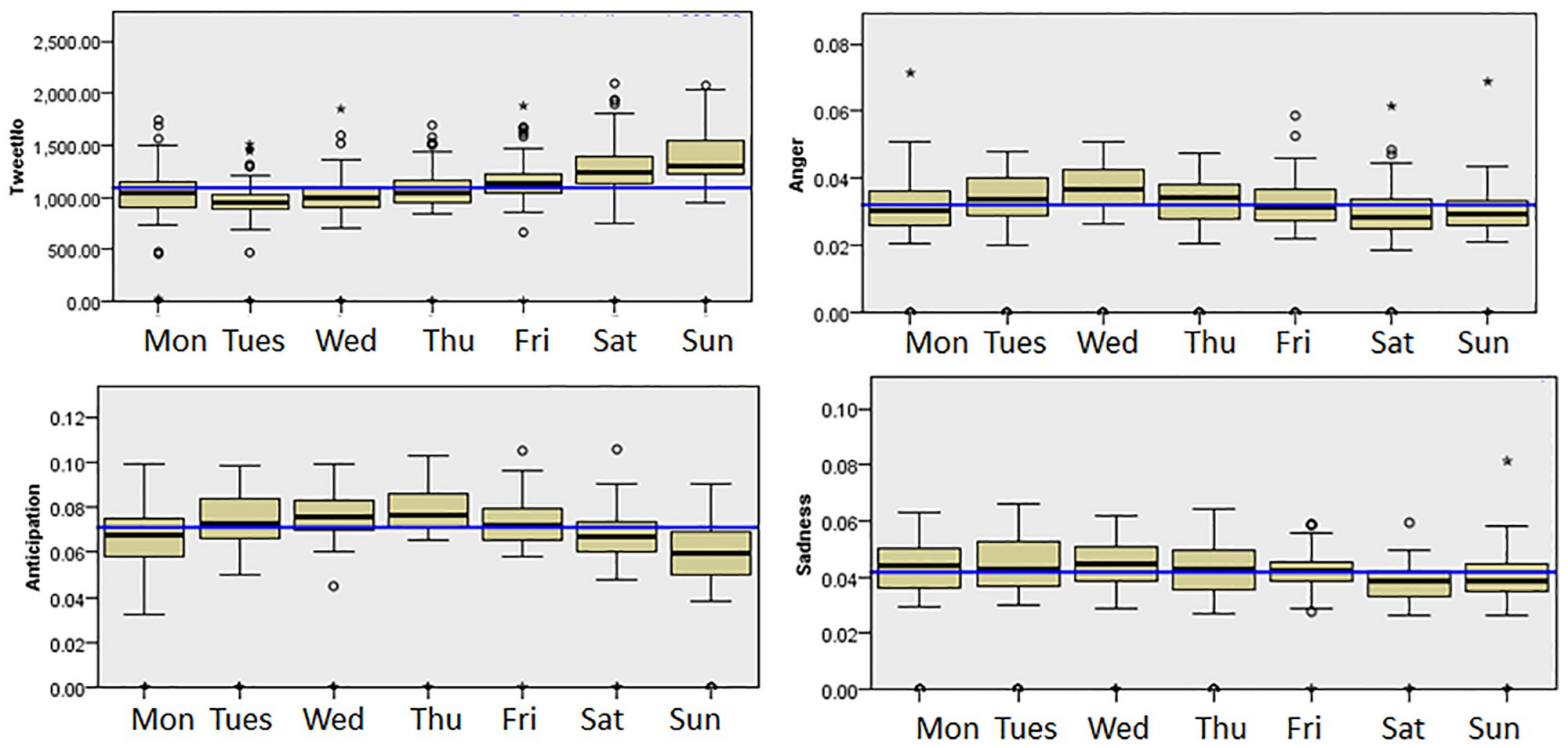
The emotions displayed on different days of the week in San Francisco.

Next, we examined the differences in the display of emotions on different days of the week. Kruskal-Wallis tests were carried out to investigate these differences. The results showed significant differences in Anger, Anticipation, Sadness in San Francisco for different days of the week (p<0.01). A visual inspection suggested that Anger was higher during the middle of the week (on Wednesday (median = 0.037)) and less during the weekends (particularly Saturday (median = 0.287) and Sunday (median = 0.029)). The least amount of Anticipation was shown on Sunday (median = 0.0596) compared to the weekdays (such as during Friday (median = 0.0722)). The weekends also tended to have a lower amount of Sadness (with a median of 0.038 on Sunday and Saturday) when compared to the weekdays (a median of 0.044 for Monday etc.). Fig 5 shows the emotions displayed on each days of the week which were significantly different in San Francisco.

**Fig 6:**
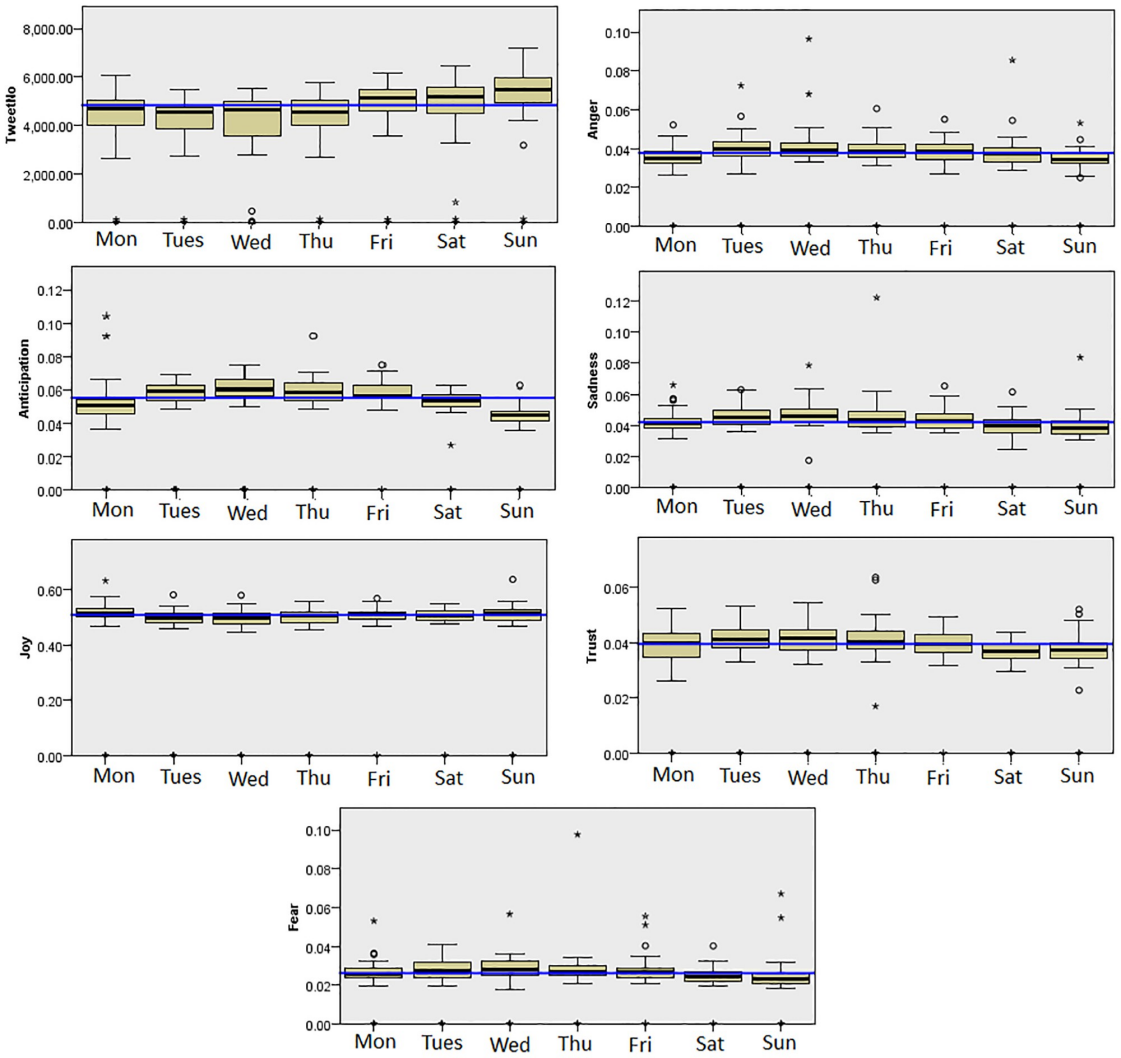
The emotions displayed on different days of the week in London.

The results were similar in London. There were significant differences in the display of Anger, Anticipation, Joy, Sadness, Fear and Trust (p<0.01). Anger, Sadness and Fear was generally lowest during the weekends, on Sunday (median Anger = 0.034, median Sadness = 0.038, median Fear = 0.023) and highest during the weekdays (Tuesday for Anger (median = 0.04), Wednesday for Sadness (median = 0.046) and Fear (median = 0.028)). Similar to San Francisco, Anticipation was also lowest during Sunday (median = 0.045 compared to an average median of 0.055 for the other days). Finally Joy was generally high on Sunday (median = 0.513), compared to an average median of 0.508 for the other days. Fig 6 shows the emotions displayed on each days of the week which were significantly different in London.

Overall, the results for both San Francisco and London could be understood quite intuitively. Positive emotions tended to be higher on weekends and negative emotions higher on weekdays [45]. This is not surprising as people have more free time to spend on Joyful activities during the weekends. Higher levels of negative emotions during Tuesday and Wednesday could perhaps be explained by people trying to get through the middle of a working week [44]. Finally, the lower amount of Anticipation on Sunday might indicate that people were not looking forward to the coming work week compared to Thursday and Friday where people were looking forward to the weekends.

## Contextualising emotions by places

In this section, we investigate the potential effect different places (based on the points of interests extracted using Open Street Map) have on the expression of emotions.

### Expression of emotions at different POIs

To analyze the effect different places have on fine-grained emotions, we examined the tweets which were near to different Points of Interests. Due to the nature of this research which explores the possible relationship between POI types and emotions, we considered tweets which were within a specific distance from a POI to fall under the influence of that POI. For each POI in our database, an appropriate radius for which a tweet could be considered to be influenced by that POI was determined by calculating the distance between each POI element in our database and the nearest non-tagged building or tagged POI (0.44 million locations in London and 0.28 million locations in San Francisco). For POI locations which were isolated, the average distance calculated from all the other POIs was used instead (which for San Francisco was 14 meters and London 9 meters). It should be noted that a POI was considered isolated when the distance to the nearest non-tagged building or tagged POI was more than the far-out outliers value (calculated using Tukey’s Fences which resulted in a distance of 51.5 meters for San Francisco and 50.0 meters for London). The distances between the tweets and the POI were calculated using the Haversine formula. Overall, there were 4,674 POIs (21.42%) which had nearby tweets from at least one Twitter user in San Francisco and 26,740 POIs (11.31%) in London.

Table 3 shows the top 10 POIs (based on the original OSM tag values) where nearby users expressed the highest level of each emotion in the city of San Francisco and London. When calculating the top 10 results, POIs with less than 10 users were excluded as well as those with only one venue in the city (as these locations tended to have artificially high averages due to a low amount of users posting few tweets which happened to be classified as positive for certain emotions). From the results, it seems that locations such as hospitals, dentist and doctor offices tend to have tweets with high amounts of fear and sadness (perhaps indicating that people are fearful for their illness or for their love ones) and locations where water related activities can be carried out such as swimming pools, sailing ports, coastlines and boat ramps showed tweets with high levels of Joy. High negative emotions such as Anger and Disgust were also shown nearby Transportation locations such as bus stops, bridges and train stations, perhaps reflecting peoples’ frustration at waiting for their transportation or being stuck in traffic.

**Table 3:** The top 10 POI for each emotion for the cities of San Francisco and London.

**San Francisco**						
**Anger**	**Anticipation**	**Disgust**	**Fear**	**Joy**	**Sadness**	**Trust**
civic service	embassy	estate agent	church	furniture	coffee	historic bunker
house	medical service	doityourself	hospital	swimming pool	erotic	historic ship
pet	luxury bags	insurance	woods	picnic site	historic ship	cliff viewpoint
greengrocers	historic ship	historic bunker	dentist	cliff viewpoint	greengrocer	coastline
doityourself	insurance	gallery	second hand	strip club	food court	recycling
computer	college	civic service	beach	doityourself	wine	gift
stationery	jewelry	greengrocers	hardware	boat ramp	fountain	ngo
repair	arts center	erotic	furniture	deli	pharmacy	taxi
gift	police	hostel	doctors	coastline	hospital	civic service
laundry	school	train station	ngo	arts center	hardware store	construction
**London**						
**Anger**	**Anticipation**	**Disgust**	**Fear**	**Joy**	**Sadness**	**Trust**
bus stop	funeral directors	bridge	locksmith	motel	bridge	co working space
spa	academic society	design agency	pier	event venue	boxing	organic market
boxing	historic gate	boxing	rugby union	home showroom	music venue	city walls
apartment	apartment	apartment	police	miniature golf	ruins	casino
skateboard	boxing	train station	embassy	guest house	garage	motel
parking	sailing	political party	bridge	sailing port	market	car wash
bridge	internet cafe	carpet	wine	farm	hospital	shop
fire station	clinic	club house	barracks	shoe repair	pier	shoe maker
barracks	dormitory	kiosk	city walls	money transfer	shop	guest house
massage	employment agency	strip club	hospital	co working space	parking	jewellery

**Fig 7:**
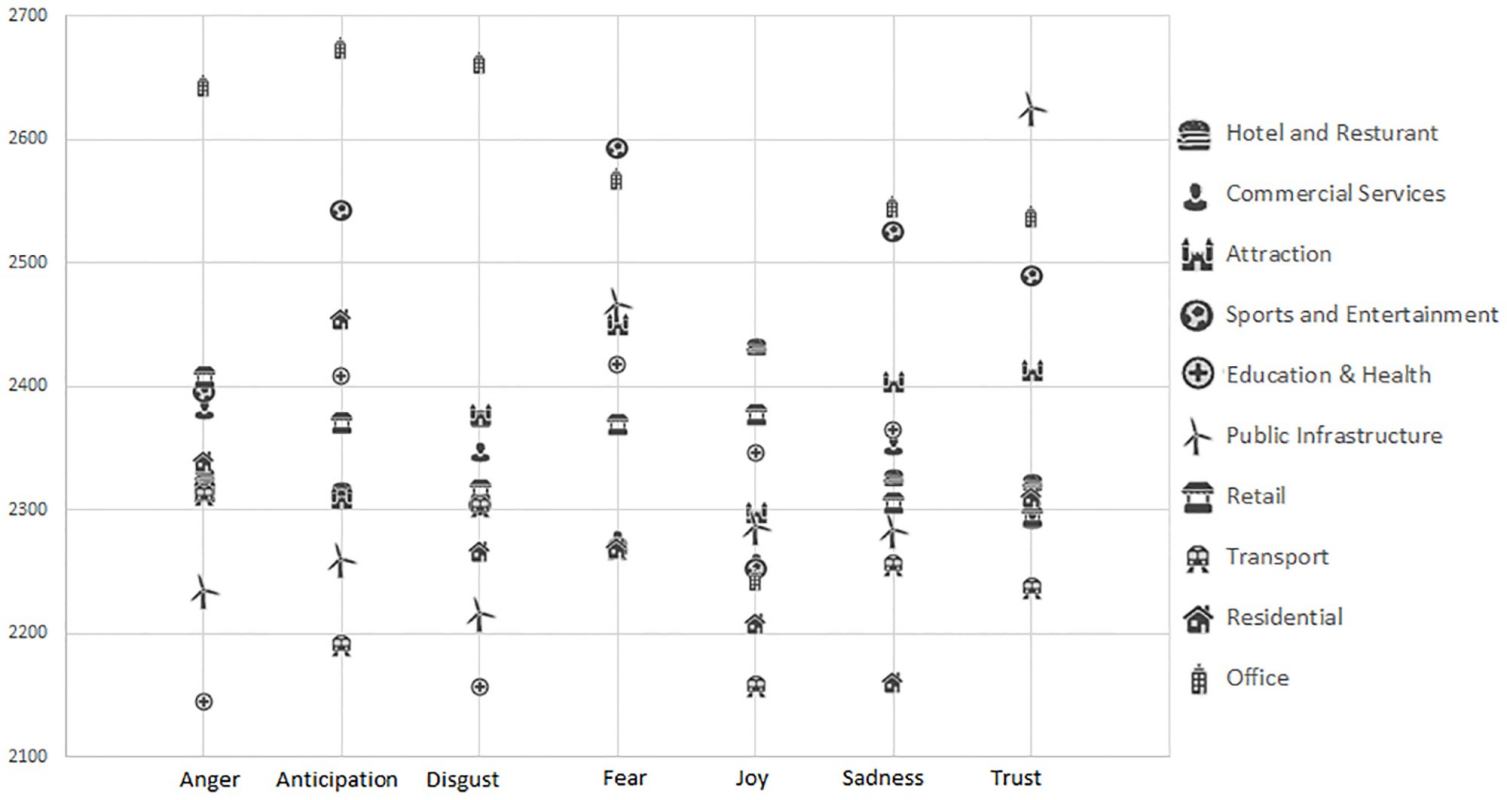
The emotions displayed for different location categories in San Francisco (based on the mean rank. A higher rank denotes a higher level of emotion).

**Fig 8:**
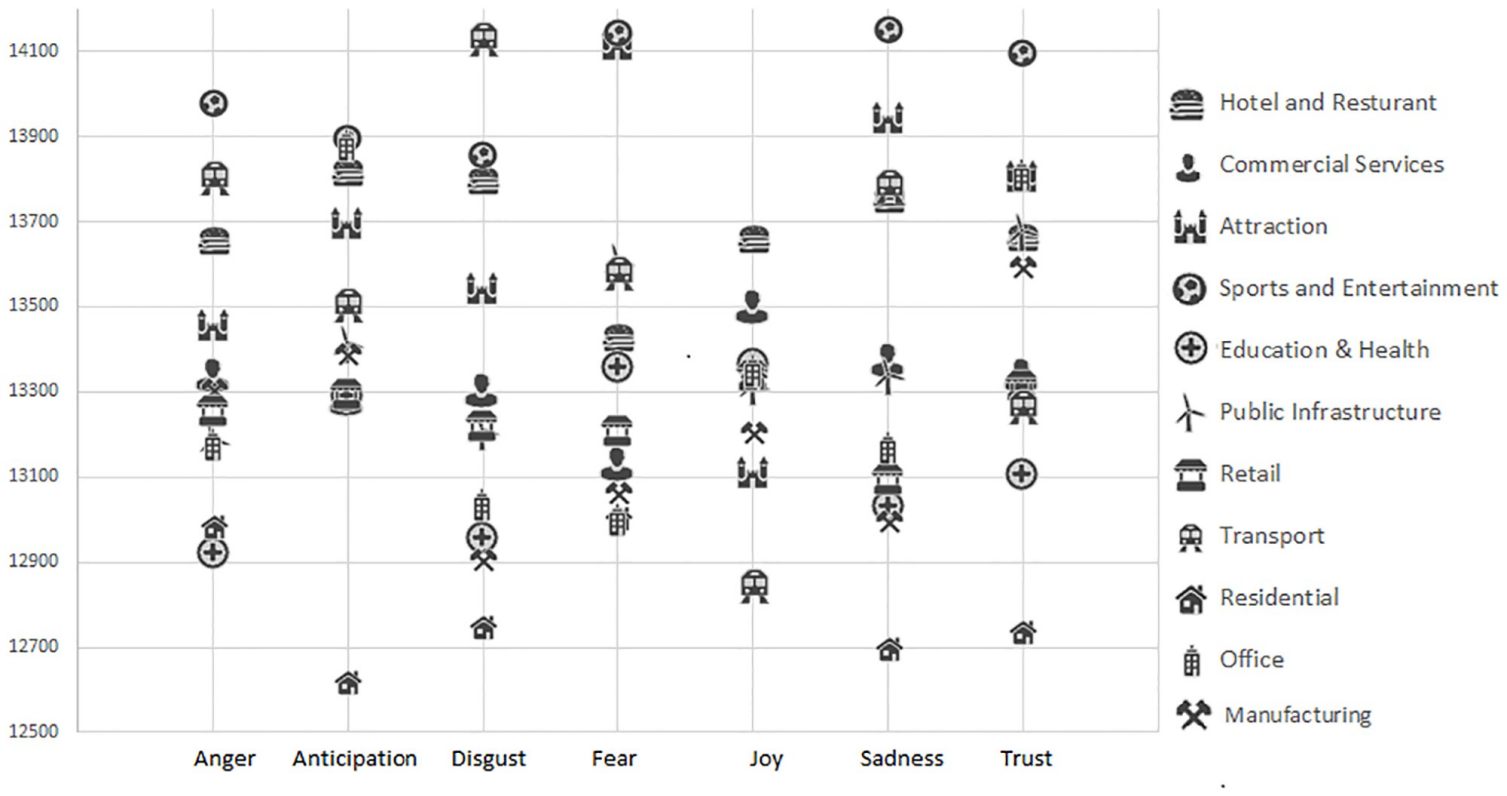
The emotions displayed for different location categories in London (based on the mean rank. A higher rank denotes a higher level of emotion).

Next, we examined the display of emotions at different POIs based on the Ordnance Survey classification values. As the distribution of the tweets in each POI location followed a pattern which was similar to a power law distribution (e.g. in San Francisco, the top 10 POI locations had on average 2,950 users tweeting and at the tail end, 29% of locations had 1 user), we had used non-parametric approaches to analyze the data in our study. More specifically, Kruskal-Wallis tests were carried to examine the differences between the emotions expressed at the different categorical locations. The results showed significant differences for all the emotions in London (p<0.05 for Joy, p<0.001 for all the other 6 emotions) and for Anger, Joy, Sadness, (p<0.05) Anticipation, Fear and Trust (p<0.01) in San Francisco. There was no significant differences between the different categories for Disgust in SF. Fig 7 shows the level of the emotions at different POI categories for San Francisco and Fig 8 shows the level of the emotions at different POI categories for London based on the mean rank obtained from the Kruskal-Wallis tests. It should be noted that the manufacturing category was excluded in the analyzed for San Francisco, due to the low number of such locations (n = 12).

Table 4 provides an overview of the significantly different categories based on the results of Bonferroni corrected Dunn’s post-hoc tests carried out following the Kruskal-Wallis tests (at a p<0.05 level). For example, the results showed that in San Francisco, there was significantly more Anger near (9) Retail and (12) Office locations than close to (5) Education & Health locations. In addition there was significantly more Joy near (1) Hotel & Restaurant locations than (10) Transportation places (bus stops etc.). In London, the results showed for example that there was significantly more Anger near (4) Sports & Entertainment, (1) Hotel and Restaurant and (10) Transportation locations than near (5) Education & Health, (11) Residential and (9) Retail locations. It should be noted that the majority of the significant differences in London was due to the (11) Residential category, where there tended to be on average, significantly less emotions exhibited on the tweets at such locations than at other locations.

**Table 4:** Results of significantly different categories: (1): Hotel & Restaurants, (2): Commercial Services, (3): Attractions, (4): Sports & Entertainment, (5): Education & Health, (6): Public Infrast., (7): Manufacturing & Production, (9): Retail, (10): Transport, (11): Residential, (12): Office.

Emotion	San Francisco	London
Anger	(9),(12) > (5)	(1),(10),(4) > (5),(11),(9)
Disgust	N/A	(1),(2),(3),(4),(9),(10) > (11) and (1),(10) > (5) and (10) > (9),(2),(6) and (1) > (9),(2)
Anticipation	(4) > (10)	(1),(2),(3),(4),(5),(6),(9), (10),(12) > (11) and (1) > (2),(9)
Joy	(1) > (10)	(1) > (10)
Sadness	(4) > (11)	(1),(2),(3),(4),(10) > (11) and (1),(3),(4),(10) > (5),(9) and (3),(4) > (2)
Fear	(4) > (1),(2),(10) and (3) > (1)	(3),(4) > (1),(2),(5),(9),(12) and (1),(3),(4),(6),(10) > (11) and (3) > (7),(10)
Trust	(6) > (10)	(1),(2),(3),(4),(6),(9),(10),(12) > (11) and (3),(4) > (5),(9) and (4) > (2),(10)

Overall, this analysis of fine-grained emotions at different POI categories provided a number of interesting findings. Similar to an earlier study which analyzed tweets at New York city, we found that transportation locations such as train stations, bus garages and bridges in London and San Francisco tend to elicit negative emotions (lower Joy and Higher Disgust etc.) [10]. Sports and Entertainment venues (such as stadiums) also tended to show high levels of Anger, Fear, Anticipation and Sadness. This is understandable as these were emotions which tended to be associated with the participation and viewing of sport activities (being fearful and sad when the team they are supporting is losing [46]. The high levels of Joy in the Hotel & Restaurant category could perhaps be explained by people tweeting about their food and tourist tweeting about their holiday. A manual inspection showed that several restaurants types in San Francisco and tourist accommodation types (motel, hostels) in London had high levels of Joy.

### POI neighborhood effect on emotions

Next, we further examine whether the display of emotions through the tweets was affected by the type of POI within the nearby vicinity. This was done by drawing a radius around the center of each positively and not positively classified tweets for each emotion and identifying the amount of venues in each category which exist in that vicinity. Distance levels of 10, 30 and 50 meters were examined for the tweets in San Francisco in this study. For example, if a positively classified Anger tweet contains 2 Restaurants, 6 Residential buildings and an Office and no other venues within 20 meters, then the independent variables for that tweet would be represented as (2,0,0,0,0,0,0,0,0,6,1) (the first representing the number of venues in the (1) Hotel & Restaurant category and the second, the number of venues in the (2) Commercial services category and so on).

Mann-Whitey tests were then carried out to examine the differences between the number of venues in each category that exist in the nearby vicinity of the tweets classified as positive and negative for each emotion. In short, the results would indicate whether the tweets which were classified as positive or negative based on a specific emotion (such as Anger), might contain significantly higher or lower numbers of venues of a specific category within the nearby vicinity. Bonferroni correction was also applied to reduce the likelihood of finding significant results by chance due to multiple comparisons.

The categories of locations which showed significant differences (p<0.05) in the display of emotions at the three different distance levels are shown in Table 5. A category number in the “More” column indicates that tweets which were positively classified based on a specific emotion tend to have a significantly higher number of venues of such category in the nearby vicinity than those which were not positively classified. A category number in the “Less” column indicates that tweets which were positively classified tend to have a significantly lower number of such venues in the nearby vicinity. For example, tweets which were positively classified as expressing Joy tend to have significantly more (1) Hotel & Restaurant, (2) Commercial Services and (4) Sports & Entertainment places within the vicinity (which might be indicative of tourist or shopping areas) than those which were not. Meanwhile, tweets which were classified as expressing Joy tend to have significantly less (12) Office locations within a 10 meter vicinity than those which were not. In addition, Angry and Sad tweets tend to have significantly higher numbers of (12) Offices in the nearby vicinity regardless of distance. There also seems to be an effect of distance in some cases, for example, Trust tweets tended to contain significantly higher numbers of (6) Public Infrastructure locations within 10 and 20 meters but a difference was no longer observed after extending the distance to 30 meters.

**Table 5:** The categories of locations which showed significant differences (p<0.05) in the display of emotions at three different distance levels in San Francisco.

Emotion	More (10m)	Less (10m)	More (20m)	Less (20m)	More (30m)	Less (30m)
Anger	(12)	(1)-(4),(9),(10)	(1),(12)	(3),(4),(9),(10)	(1),(9),(12)	(2)-(4),(6)
Anticipation	(4),(12)	(1),(3)	(2),(4),(12)	(3),(10)	(2),(4),(12)	(3),(10)
Disgust	(12)	(1)	(5),(12)	(3),(4)	(9),(11),(12)	(3),(4)
Fear	(3),(6)	(1)	(2),(3),(5)	(1),(4)	(2),(3),(5)	(1),(4)
Joy	(1),(4)	(12)	(2),(4)	(3),(5),(6),(9)-(12)	(2)	(1),(3),(5)-(7),(9)-(12)
Sadness	(12)	(1)	(3),(4),(12)	(1),(4),(9)	(3),(12)	(1),(4),(6)
Trust	(6),(10)	(1),(12)	(6),(10)	(1),(12)	(7),(10)	(1),(9),(12)

## Conclusions & future work

In this paper, open web data from Twitter and Open Street Map was analyzed to portray fine-grained human emotions in two cities, San Francisco and London. Neural Network classifiers were developed, tested and used to classify approximately 2 million geotagged tweets based on 7 different emotions. A temporal examination regarding the portrayal of the different emotions was provided, highlighting for example how tweets in London displayed high levels of Sadness and Fear during two high profile terrorism incidents which occurred in the city, while political protest events resulted in high levels of Anger, Disgust and Sadness in San Francisco. Afterwards, the tweets were matched to POIs extracted from Open Street Map to examine the effect of different places on the display of emotion. The results showed significant differences with regard to the type of place, such as how tweets in London showed significantly more Anger at Transportation and Sport locations than at Education, and Residential locations and how tweets which display Anger and Sadness in SF tend to have significantly higher numbers of Office venues in the nearby vicinity.

Compared to existing research, our work makes several novel contributions. While prior studies investigating the spatial distribution of emotions through social media tend to focus on sentiment polarity [9, 47] and are deployed at a coarse geographical level (state or country wide etc.) [6, 7, 18], our study shows how 7 different fine-grained emotions could be portrayed at a POI level in a finer scale. In addition, we show how our approach could be beneficial to research related to the spatial and temporal characteristics of the fine-grained emotions by providing an overview into the effect different categories of places could have on the expression of emotion. Our work thus provides a broad overview of how expressing emotions on Twitter reflects the surrounding spatial-temporal context.

Accordingly, more in-depth analyses could be carried out to examine the expression of emotions at more specific location categories (different types of restaurant (fast food, cafe etc.), recreational facilities (sports, parks, bars)). In particular, we would be interested in addressing more specific research questions, such as the degree different places might have as a buffer for extreme emotions on days with adverse events (during terrorist events, do bars help reduce Fear when compared to places such as parks? etc.). There are a number of research areas which this work could be expanded into, for example, in areas such as digital archiving (to record not only facts, but also collective human emotional responses at a detailed spatial and temporal level).

Overall, there are a number of limitations in our study. As geotagged tweets were used as the main data source and we focused our data collection on two urban English speaking cities, the results might not be generalizable to other population groups with different languages and cultures. Furthermore, previous studies have also highlighted several biases that could be present in data from geotagged tweets, such as demographical biases, where users who post geotagged tweets tend to be younger and more educated than the general population [48, 49] and female users could be overrepresented in the urban city areas where we conducted our analysis [50]. Compared with non-geotagged tweets, there could also be significant differences in the age and gender of users who chose to share their location, though the effect of such differences tend to be small [51]. Users are also more likely to share information about specific public or social events in such tweets [52] and posting on social media in general represents a selection bias, in that individuals who may not feel elevated levels of emotion may not see a need to Tweet. As such, researchers should be cautious about overgeneralizing the results from this study to the overall population. In addition, we should also mention that due to the large amount of data, an automatic algorithm was employed to detect emotions from tweets. While we had experimented with several algorithms and selected a sufficiently high performing model with a respectable level of accuracy to use in our analysis, same as in the case of other studies which utilize an automated approach to detect emotions, there could still be errors in the classification process which could influence the results.

Finally, we should note that as the method which we used in this study relied primarily on VGI and Social media data, our method might not be as applicable to locations with a small digital footprint. For example, in the UK, the data from OpenStreetMap of Northumberland (a county which is about 3.3 times larger than the Greater London area, but has a far lower population density (64 people per square kilometers vs 5,671 for the Greater London area) contains mostly residential locations and few or no POIs of certain types (e.g. only 2 college and university tagged POIs vs 546 for London and no POIs tagged as “motorcycle parking”, “car sharing” and “jewelry” for Northumberland). In addition, there are also fewer recorded locations even for POI types commonly found in dense cities (e.g. 99 parks vs 2,953 in London, 135 retail vs 4,280 or 2 dry cleaning vs 435 in London). When querying geo-tagged tweets which fall under the Northumberland area, far less usable tweets were also identified for the area (approximately 34,000 geotagged tweets compared with 0.6 million for London in 2021). This results in fewer tweets being matched to the POI locations, making it not as practical to conduct the type of fine-grained POI-based statistical analysis which we had done in this study (as sparse data could bias the results). Other studies of this nature which utilized social media and VGI data as the data source have also highlighted similar limitations [53] (e.g. geo-tagged social media and VGI data tend to concentrate in large metropolitan cities and while they are accurate and comprehensive in such areas, they are less present and could be less accurate in smaller rural areas and cities [24, 54, 55]. Despite these limitations, we believe that the approach used in this paper would still provide valuable insight as it allows for a large scale analysis of fine-grained emotions through openly available web data and could serve as the basis of future research in this direction.

## Supporting information

S1 Fig The structure of the hybrid Neural Network model. (TIF)

S2 Fig A time series display of emotions for San Francisco. (TIF)

S3 Fig A time series display of emotions for London. (TIF)

## References

[1] Ansari MZ, Aziz M, Siddiqui M, Mehra H, Singh K. Analysis of political sentiment orientations on twitter. Procedia Computer Science. 2020;167:1821–1828. doi: 10.1016/j.procs.2020.03.201

[2] Choi HJ, Park CH. Emerging topic detection in twitter stream based on high utility pattern mining. Expert systems with applications. 2019;115:27–36. doi: 10.1016/j.eswa.2018.07.051

[3] Hasan S, Zhan X, Ukkusuri SV. Understanding urban human activity and mobility patterns using large-scale location-based data from online social media. In: Proceedings of the 2nd ACM SIGKDD international workshop on urban computing. ACM; 2013. p. 6.

[4] Terroso-Saenz F, Muñoz A, Arcas F, Curado M. Can Twitter be a Reliable Proxy to Characterize Nation-wide Human Mobility? A Case Study of Spain. Social Science Computer Review. 2022; p. 08944393211071071. doi: 10.1177/08944393211071071

[5] Mislove A. Pulse of the nation: US mood throughout the day inferred from Twitter; 2010. Available from: http://www.ccs.neu.edu/home/amislove/twittermood/.

[6] Ashkezari-Toussi S, Kamel M, Sadoghi-Yazdi H. Emotional maps based on social networks data to analyze cities emotional structure and measure their emotional similarity. Cities. 2019;86:113–124. doi: 10.1016/j.cities.2018.09.009

[7] Pauken B, Pradyumn M, Tabrizi N. Tracking happiness of different US cities from tweets. In: International conference on Big Data. Springer; 2018. p. 140–148.

[8] Mitchell L, Frank MR, Harris KD, Dodds PS, Danforth CM. The geography of happiness: Connecting twitter sentiment and expression, demographics, and objective characteristics of place. PloS one. 2013;8(5):e64417. doi: 10.1371/journal.pone.0064417 23734200 PMC3667195

[9] Cao X, MacNaughton P, Deng Z, Yin J, Zhang X, Allen JG. Using twitter to better understand the spatiotemporal patterns of public sentiment: A case study in Massachusetts, USA. International journal of environmental research and public health. 2018;15(2):250. doi: 10.3390/ijerph15020250 29393869 PMC5858319

[10] Bertrand KZ, Bialik M, Virdee K, Gros A, Bar-Yam Y. Sentiment in new york city: A high resolution spatial and temporal view. arXiv preprint arXiv:13085010. 2013;.

[11] Valdez D, Ten Thij M, Bathina K, Rutter LA, Bollen J, et al. Social media insights into US mental health during the COVID-19 pandemic: Longitudinal analysis of Twitter data. Journal of medical Internet research. 2020;22(12):e21418. doi: 10.2196/21418 33284783 PMC7744146

[12] Kumar V. Spatiotemporal sentiment variation analysis of geotagged COVID-19 tweets from India using a hybrid deep learning model. Scientific Reports. 2022;12(1):1–14. doi: 10.1038/s41598-022-05974-6 35115652 PMC8814057

[13] Dai D, Wang R. Space-time surveillance of negative emotions after consecutive terrorist attacks in London. International journal of environmental research and public health. 2020;17(11):4000. doi: 10.3390/ijerph17114000 32512901 PMC7313064

[14] Gallegos L, Lerman K, Huang A, Garcia D. Geography of Emotion: Where in a City are People Happier? In: Proceedings of the 25th International Conference Companion on World Wide Web. International World Wide Web Conferences Steering Committee; 2016. p. 569–574.

[15] Guo W, Gupta N, Pogrebna G, Jarvis S. Understanding happiness in cities using Twitter: Jobs, children, and transport. In: Smart Cities Conference (ISC2), 2016 IEEE International. IEEE; 2016. p. 1–7.

[16] Kim J, Cha M, Sandholm T. Socroutes: safe routes based on tweet sentiments. In: Proceedings of the 23rd International Conference on World Wide Web. ACM; 2014. p. 179–182.

[17] Chen X, Cho Y, Jang SY. Crime prediction using Twitter sentiment and weather. In: 2015 Systems and Information Engineering Design Symposium. IEEE; 2015. p. 63–68.

[18] Larsen ME, Boonstra TW, Batterham PJ, O’Dea B, Paris C, Christensen H. We feel: mapping emotion on Twitter. IEEE journal of biomedical and health informatics. 2015;19(4):1246–1252. doi: 10.1109/JBHI.2015.2403839 25700477

[19] Guthier B, Alharthi R, Abaalkhail R, El Saddik A. Detection and visualization of emotions in an affect-aware city. In: Proceedings of the 1st International Workshop on Emerging Multimedia Applications and Services for Smart Cities. ACM; 2014. p. 23–28.

[20] Lin YR, Margolin D, Wen X. Tracking and analyzing individual distress following terrorist attacks using social media streams. Risk analysis. 2017;37(8):1580–1605. doi: 10.1111/risa.12829 28556273

[21] Reyes-Riveros R, Altamirano A, De la Barrera F, Rozas-Vasquez D, Vieli L, Meli P. Linking public urban green spaces and human well-being: A systematic review. Urban Forestry & Urban Greening. 2021;61:127105. doi: 10.1016/j.ufug.2021.127105

[22] Hogertz C. Emotions of the urban pedestrian: sensory mapping. Pedestrians quality needs. 2010;31.

[23] Resch B, Summa A, Sagl G, Zeile P, Exner JP. Urban emotions Geo semantic emotion extraction from technical sensors, human sensors and crowdsourced data. In: Progress in location-based services 2014. Springer; 2015. p. 199–212.

[24] Haklay M. How good is volunteered geographical information? A comparative study of OpenStreetMap and Ordnance Survey datasets. Environment and planning B: Planning and design. 2010;37(4):682–703. doi: 10.1068/b35097

[25] OpenStreetMap. Open Street Map; 2022. https://www.openstreetmap.org/.

[26] Zheng S, Zheng J. Assessing the completeness and positional accuracy of OpenStreetMap in China. In: Thematic cartography for the society. Springer; 2014. p. 171–189.

[27] Helbich M, Amelunxen C, Neis P, Zipf A. Comparative spatial analysis of positional accuracy of OpenStreetMap and proprietary geodata. Proceedings of GI_Forum. 2012; p. 24–33.

[28] Ordnance Survey GB. Points of interest classification scheme; 2021. https://www.ordnancesurvey.co.uk/business-government/tools-support/points-of-interest-support.

[29] Open Street Map Wiki. Tags-Open Street Map Wiki; 2022. https://wiki.openstreetmap.org/wiki/Tags.

[30] Kruspe A, Häberle M, Hoffmann EJ, Rode-Hasinger S, Abdulahhad K, Zhu XX. Changes in Twitter geolocations: Insights and suggestions for future usage. arXiv preprint arXiv:210812251. 2021;.

[31] Plunz RA, Zhou Y, Vintimilla MIC, Mckeown K, Yu T, Uguccioni L, et al. Twitter sentiment in New York City parks as measure of well-being. Landscape and urban planning. 2019;189:235–246. doi: 10.1016/j.landurbplan.2019.04.024

[32] Yang C, Srinivasan P. Life satisfaction and the pursuit of happiness on Twitter. PloS one. 2016;11(3):e0150881. doi: 10.1371/journal.pone.0150881 26982323 PMC4794168

[33] Branz L, Brockmann P. Sentiment analysis of twitter data: towards filtering, analyzing and interpreting social network data. In: Proceedings of the 12th ACM International Conference on Distributed and Event-based Systems; 2018. p. 238–241.

[34] Da Silva NF, Hruschka ER, Hruschka ER Jr. Tweet sentiment analysis with classifier ensembles. Decision Support Systems. 2014;66:170–179. doi: 10.1016/j.dss.2014.07.003

[35] Mohammad S. Portable Features for Classifying Emotional Text. In: Proceedings of the 2012 Conference of the North American Chapter of the Association for Computational Linguistics: Human Language Technologies. NAACL HLT’12. Stroudsburg, PA, USA: Association for Computational Linguistics; 2012. p. 587–591. Available from: http://dl.acm.org/citation.cfm?id = 2382029.2382123.

[36] Plutchik R. The nature of emotions: Human emotions have deep evolutionary roots, a fact that may explain their complexity and provide tools for clinical practice. American scientist. 2001;89(4):344–350. doi: 10.1511/2001.4.344

[37] Pennington J, Socher R, Manning CD. GloVe: Global Vectors for Word Representation. In: Empirical Methods in Natural Language Processing (EMNLP); 2014. p. 1532–1543. http://www.aclweb.org/anthology/D14-1162.

[38] Bravo-Marquez F, Frank E, Mohammad SM, Pfahringer B. Determining word-emotion associations from tweets by multi-label classification. In: Web Intelligence (WI), 2016 IEEE/WIC/ACM International Conference on. IEEE; 2016. p. 536–539.

[39] Agarwal A, Toshniwal D. Application of lexicon based approach in sentiment analysis for short tweets. In: 2018 International Conference on Advances in Computing and Communication Engineering (ICACCE). IEEE; 2018. p. 189–193.

[40] Kudugunta S, Ferrara E. Deep Neural Networks for Bot Detection. arXiv preprint arXiv:180204289. 2018;.

[41] Tukey JW. Exploratory data analysis. vol. 2. Reading, Mass.; 1977.

[42] Lin YR, Margolin D. The ripple of fear, sympathy and solidarity during the Boston bombings. EPJ Data Science. 2014;3(1):31. doi: 10.1140/epjds/s13688-014-0031-z

[43] Curini L, Iacus S, Canova L. Measuring idiosyncratic happiness through the analysis of twitter: An application to the italian case. Social Indicators Research. 2015;121(2):525–542. doi: 10.1007/s11205-014-0646-2

[44] Dodds PS, Harris KD, Kloumann IM, Bliss CA, Danforth CM. Temporal patterns of happiness and information in a global social network: Hedonometrics and Twitter. PloS one. 2011;6(12):e26752. doi: 10.1371/journal.pone.0026752 22163266 PMC3233600

[45] Trampe D, Quoidbach J, Taquet M. Emotions in everyday life. PloS one. 2015;10(12):e0145450. doi: 10.1371/journal.pone.0145450 26698124 PMC4689475

[46] Yu Y, Wang X. World Cup 2014 in the Twitter World: A big data analysis of sentiments in US sports fans’ tweets. Computers in Human Behavior. 2015;48:392–400. doi: 10.1016/j.chb.2015.01.075

[47] Li M, Ch’ng E, Chong A, See S. The new eye of smart city: Novel citizen Sentiment Analysis in Twitter. In: Audio, Language and Image Processing (ICALIP), 2016 International Conference on. IEEE; 2016. p. 557–562.

[48] Blank G, Lutz C. Representativeness of social media in great britain: investigating Facebook, Linkedin, Twitter, Pinterest, Google+, and Instagram. American Behavioral Scientist. 2017;61(7):741–756. doi: 10.1177/0002764217717559

[49] Smith A, Anderson M. Social Media Use in 2018. Pew Research Report. 2018;.

[50] Jiang Y, Li Z, Ye X. Understanding demographic and socioeconomic biases of geotagged Twitter users at the county level. Cartography and geographic information science. 2019;46(3):228–242. doi: 10.1080/15230406.2018.1434834

[51] Sloan L, Morgan J. Who tweets with their location? Understanding the relationship between demographic characteristics and the use of geoservices and geotagging on Twitter. PloS one. 2015;10(11):e0142209. doi: 10.1371/journal.pone.0142209 26544601 PMC4636345

[52] Nguyen QC, Li D, Meng HW, Kath S, Nsoesie E, Li F, et al. Building a national neighborhood dataset from geotagged Twitter data for indicators of happiness, diet, and physical activity. JMIR public health and surveillance. 2016;2(2):e5869. doi: 10.2196/publichealth.5869 27751984 PMC5088343

[53] Wakamiya S, Kawai Y, Aramaki E, et al. Twitter-based influenza detection after flu peak via tweets with indirect information: text mining study. JMIR public health and surveillance. 2018;4(3):e8627. doi: 10.2196/publichealth.8627 30274968 PMC6231889

[54] Hecht B, Stephens M. A tale of cities: Urban biases in volunteered geographic information. In: proceedings of the international AAAI conference on web and social media. vol. 8; 2014. p. 197–205.

[55] Carley KM, Malik M, Landwehr PM, Pfeffer J, Kowalchuck M. Crowd sourcing disaster management: The complex nature of Twitter usage in Padang Indonesia. Safety science. 2016;90:48–61. doi: 10.1016/j.ssci.2016.04.002

