# Supplementary figures and images for: A city-wide examination of fine-grained human emotions through social media analysis
Source: PLoS One. 2023 Feb 1;18(2):e0279749. doi: 10.1371/journal.pone.0279749 (PMC9891511; doi:10.1371/journal.pone.0279749)

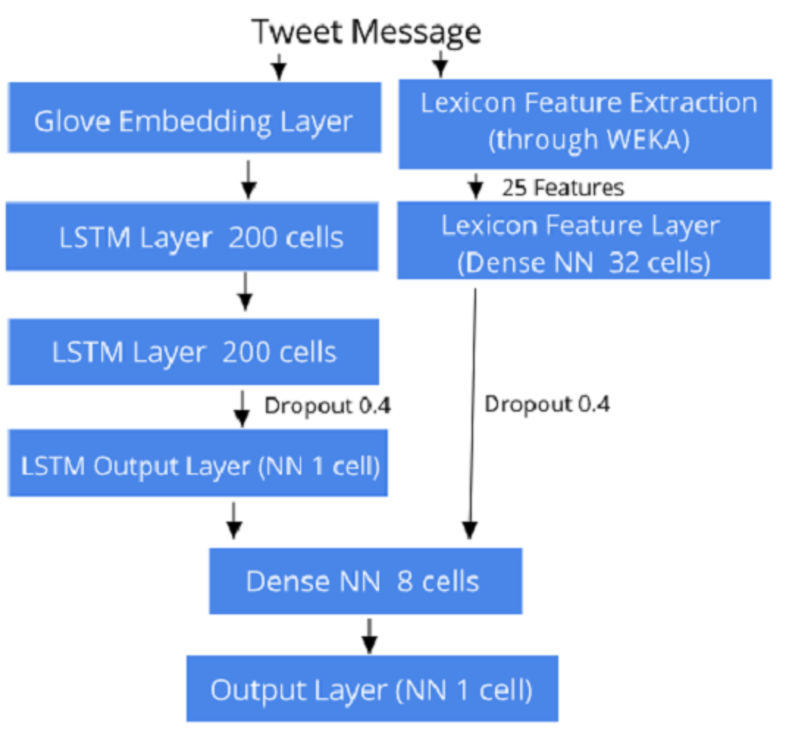

Supplement: S1 Fig — (TIF) [file pone.0279749.s001.tif]

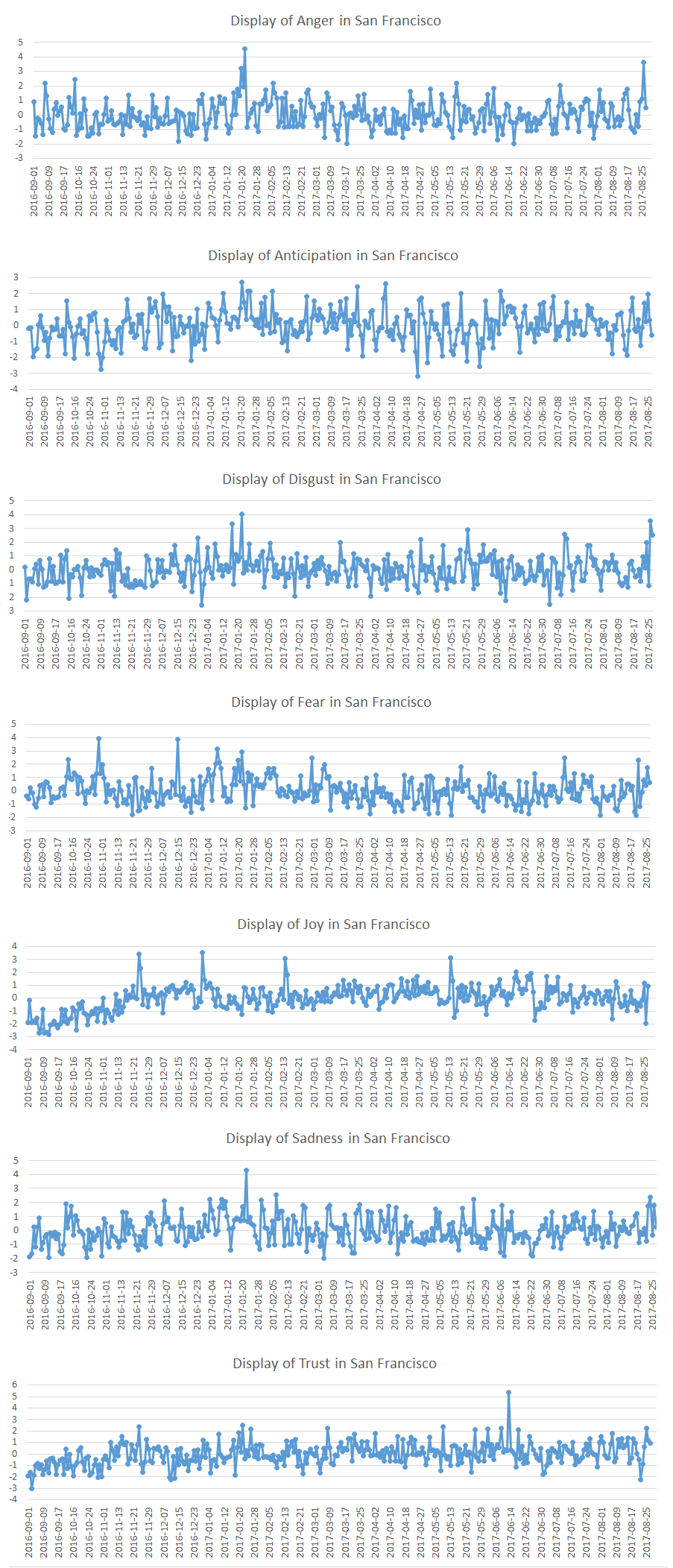

Supplement: S2 Fig — (TIF) [file pone.0279749.s002.tif]

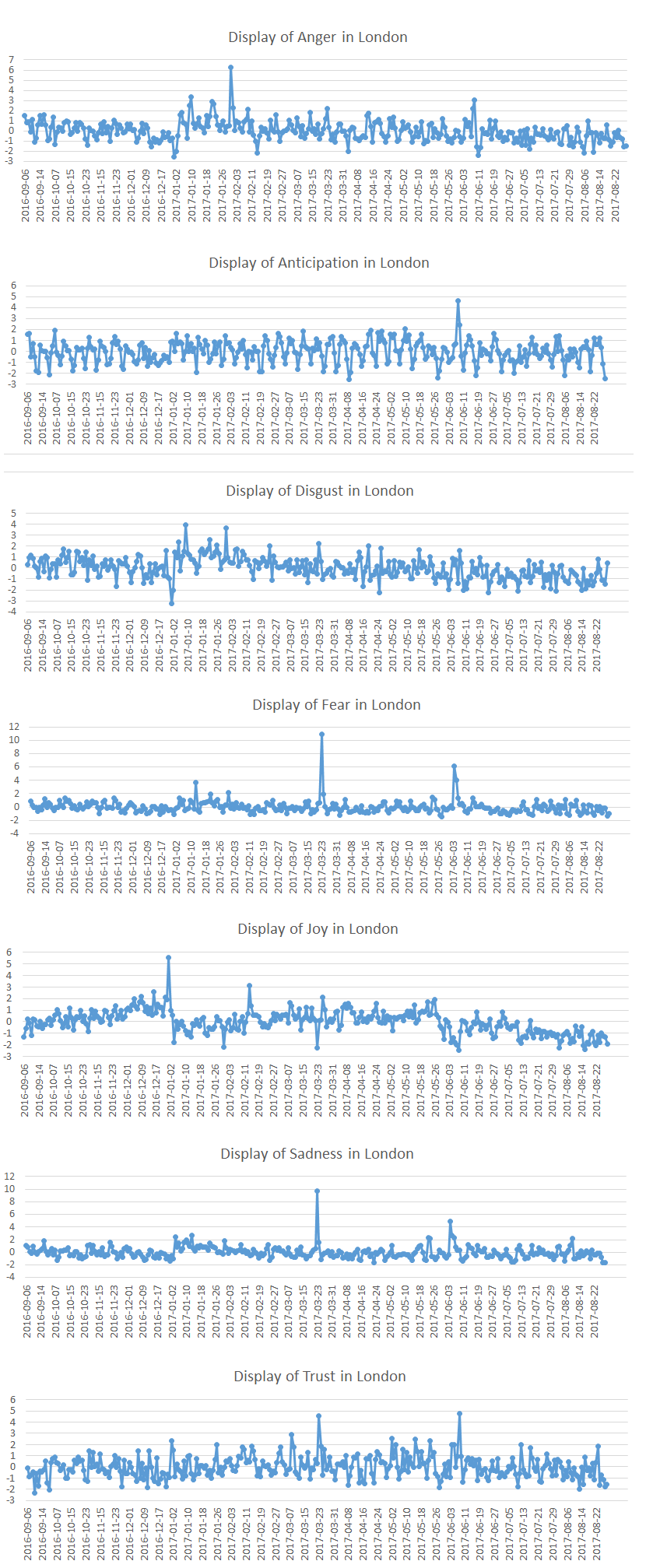

Supplement: S3 Fig — (TIF) [file pone.0279749.s003.tif]
